# Primary Prevention of First-Ever Stroke in Primary Health Care: A Clinical Practice Study Based on Medical Register Data in Sweden

**DOI:** 10.4061/2010/468412

**Published:** 2010-07-11

**Authors:** Ylva Skånér, Gunnar H. Nilsson, Ingvar Krakau, Ejda Hassler, Kristina Sundquist

**Affiliations:** Department of Neurobiology, Care Sciences and Society, Center for Family and Community Medicine, Karolinska Institutet, Alfred Nobels allé 12, 141 83 Huddinge, Sweden

## Abstract

*Background*. The aim of this study was to investigate whether established risk factors for stroke in patients admitted to health care for first-ever stroke had been detected and treated in primary health care. *Methods*. In a retrospective study in Nacka municipality, Stockholm County, Sweden, with about 70 000 inhabitants, we included all men and women admitted to health care due to first-ever stroke between October 1999 and March 2001. Data on 187 such patients, with a mean age of 75 years, were obtained from medical registers. Main outcome measures were detection and treatment of risk factors for stroke including hypertension, diabetes, atrial fibrillation, smoking, alcohol abuse, and overweight/obesity. *Results*. In a majority of patients seen in primary health care with hypertension and diabetes, those risk factors were detected and treated (75.6% and 75.0%, resp.). Fewer patients with atrial fibrillation received treatment (60.9%). Treatment of lifestyle factors was difficult to assess because of lack of data in the medical records. *Conclusions*. Primary prevention of stroke in primary health care needs to be improved, especially when atrial fibrillation and lifestyle-related risk factors are present. Health policies need to target not only the public, but also general practitioners and other health care professionals.

## 1. Background

Stroke is a major cause of death and disability in the ageing population of Western countries. The financial burden of stroke on society is considerable and includes high direct and indirect costs for stroke care for afflicted individuals [1]. In order to reduce these costs and improve elderly people health, it is essential to control risk factors for stroke. The major strategy for reducing stroke is therefore prevention, with the focus on the most prevalent modifiable risk factors. This is especially important in primary health care (PHC), where many patients are encountered before the occurrence of stroke. Risk factors can be described as nonmodifiable or modifiable. For example, non-modifiable risk factors include high age, male sex, and a family history of stroke [2]. Modifiable risk factors include hypertension, atrial fibrillation, elevated cholesterol levels, smoking, physical inactivity, obesity, carotid stenosis, and heavy drinking (alcoholism) [2]. In addition, good control of blood glucose levels and aggressive treatment of hypertension are recommended for patients with diabetes [3]. 

A review of randomised clinical trials showed that a decrease in diastolic blood pressure of 5-6 mm Hg reduced the risk for stroke by 42% [4]. The SPAF 1 study of atrial fibrillation and stroke was terminated when it was found, at an interim analysis, that Warfarin and Aspirin reduced primary stroke by 67% and 42%, respectively [5]. However, rhythm control does not seem to prevent stroke [6]. When people aged 60 years or older were surveyed in NHANES III, hypertension was present in 60%–70% [7]. Previous studies of prevention of stroke in PHC have rarely included multiple risk factors. 

The aim of this study was to investigate whether established risk factors for stroke in patients admitted to health care for a first-ever stroke had been detected and treated by a general practitioner in PHC. 

## 2. Methods

### 2.1. Setting

Nacka is a municipality of approximately 70 000 inhabitants situated about ten kilometres from the centre of Stockholm. We checked whether Nacka municipality was representative for the rest of Sweden by measuring the area-based demographic and socioeconomic characteristics, which were defined by the Care Need Index. This is an area-based index aimed for use in distributing PHC resources [8]. Calculation of the Care Need Index resulted in a value of 0.63 for Nacka as a whole, that is, very close to the mean value in Sweden, which is 0 (range –53 to +79).

### 2.2. Inclusion Criteria and Search Strategies

The present study is part of a larger study aimed at including all patients who were registered residents of Nacka at the time of hospitalisation for a first-ever stroke. The 18-month inclusion period was between October 1999 and March 2001. The general aim of the larger study was to investigate different aspects of health among the patients such as comorbidity, social situation, contacts with the health care system, quality of life, and so forth [9]. In a first substudy we investigated self-rated health, symptoms of depression, and general symptoms [10]. The aim of this second study was, however, to investigate whether established risk factors for stroke had been detected and treated in PHC. 

Several overlapping sources of case ascertainment were used, in accordance with recommendations for complete, community-based case ascertainment [11]. A regular monthly search for new cases of first-ever stroke was carried out throughout the study period, and consisted of (1) searches in patient records at the regional hospitals, (2) searches in discharge summaries from the geriatric department at the municipal hospital, (3) searches in records at the radiology departments at both the regional hospital and its subsidiary at the municipal hospital for patients who had undergone any radiological investigation of the head (considered obligatory for stroke patients), (4) reports about new cases from contact persons in the project (two stroke nurses, a rehabilitation officer, and an occupational therapist), (5) reports from managers of the health care centres in Nacka (with knowledge about new cases of stroke at the nursing homes), and (6) a complementary search of the County Council's joint register of hospital care. Searches according to (2) and (6) were done by use of ICD-10 diagnoses, searches according to (1) were done by reading the medical records of all newly admitted patients, and searches according to (3) were done by reading all relevant referral notes, and if any indications of stroke were found, by reading the medical records for further information. The search was performed in the medical records obtained through the central medical register covering all hospitals in Stockholm County. The search in all medical databases was facilitated by the Swedish system of personal code number. Only patients with first-ever stroke who were living in Nacka municipality were included, that is, a total of 187 patients. 

Stroke was defined according to WHO criteria as a sudden focal disturbance of brain function with symptoms that persist for at least 24 hours, or which leads to death, and in which the cause is not obviously nonvascular. The following codes from the International Classification of Diseases (ICD-10) were included: cerebral haemorrhage (I 61), cerebral infarction (I 63), and stroke, not specified as haemorrhage or infarction (I 64). Patients with subarachnoid haemorrhage (I 60) were not included, due to differences regarding aetiology, risk factor pattern, and management. 

After the 187 patients with first-ever stroke were identified, a retrospective search was performed for each patient in the central medical register in Stockholm County. This search was carried out in order to find all health care contacts (hospital care, hospital ambulatory care, and PHC) 12 months backwards from the date of the hospitalisation for first-ever stroke. The medical records for all health care contacts for each patient were searched for the following risk factors: hypertension, diabetes, atrial fibrillation, smoking, alcoholism, and overweight/obesity. The riskfactors were chosen according to recommendations in the regional guidelines used in Stockholm at the time of the study (http://www.VISS.nu/). Data on cholesterol levels were not included, because at the time of the study, the use of medication for elevated cholesterol levels had not yet become a part of clinical practice in PHC, at least not to any great extent. 

A retrospective search was also carried out in the medical records for all PHC in the catchment area 12 months backwards from the date of the hospitalisation (or other care) for first-ever stroke. This search was performed in order to find the patients who had visited a doctor in PHC during the 12 months before the stroke occurred. A total of 114 patients were found who had visited a doctor in PHC at least once. The 114 patients' medical records were searched for the same risk factors as above, that is, hypertension, diabetes, atrial fibrillation, smoking, alcoholism, and overweight/obesity. The medical records were read carefully by one of the co-authors (E. Hassler) in order to determine whether the risk factors for stroke had been detected and treated in PHC. Some encounters dealt only with minor ailments, while others were more thorough medical examinations. The diagnoses of hypertension and diabetes were obtained from the text or from information in the medical records about medication for hypertension or diabetes. Information on atrial fibrillation was obtained from the text and/or from electrocardiograms. Information on smoking, alcoholism, and overweight/obesity was obtained from the free text. The medical records in PHC were searched for information on measures taken, such as a booked additional control and/or initiation of treatment for hypertension, change in treatment for hypertension, initiation of treatment or referral to a cardiologist for patients with atrial fibrillation, initiation, or change in treatment and/or referral to an endocrinologist for patients with diabetes, verbal information on smoking cessation or prescriptions for nicotine replacement therapy, prescriptions for antabus, and referral to a dietician. The definition of measures taken also included the doctor's judgement that no additional treatment was needed and his/her comments on that in the text, for example, when a patient with diabetes was judged to have good blood glucose control. For the patients with atrial fibrillation, CHADS score was calculated in order to evaluate the need for anticoagulation treatment (CHADS being an acronym for Congestive heart failure, Hypertension, Age > 75, Diabetes mellitus, and prior Stroke or transient ischemic attack) [12]. Patients with score 1 or more should be anticoagulated, with ASA if score 1, with ASA or warfarin if score 2, and with warfarin if score 3 or more. 

For differences between groups chi-square analysis was used.

## 3. Results

The study group consisted of 187 first-ever stroke patients, 100 (53.5%) were women; the mean age was 75 years (SD ±12), and the range was 36–92 years of age. The 114 patients who had seen a general practitioner were older than those who had not seen a general practitioner, 76.9 (95% CI 74.84–78.96) compared to 71.9 (95% CI 61.12–74.67). 

Table 1 shows the number of patients with each risk factor (based on the medical records from all health care contacts) for all the patients, for the patients who had visited a general practitioner in PHC, and for the patients who had not, and also the number of patients where some kind of measure was taken in PHC, according to each risk factor. No statistically significant differences were found between the patients who had and who had not seen a general practitioner in the year preceding their stroke, with regard to the detected proportions of these risk factors. 

The most prevalent risk factor was hypertension (41.7%). The CHADS scores for the 23 patients with atrial fibrillation who had visited a general practitioner were as follows: 1 for 10 patients (43.5%), 2 for 8 patients (34.8%), and 3 for 5 patients (21.7%). 

Among patients with hypertension seen in PHC, some kind of measure was taken in 75.6%, and the corresponding percentage for diabetes was 75.0%, and for atrial fibrillation it was 60.9%. The percentages for measures taken for risk factors associated with the patient's lifestyle (smoking, alcoholism, and overweight/obesity) shown in Table 1 seem to be rather high, but the number of missing data in the medical records was very high concerning these factors.

In an additional analysis we investigated whether there was a difference in the number of doctor's visits for the stroke patients as compared with the general population, but no significant differences were found (data not shown). 

## 4. Discussion

The main finding of this study was that primary prevention of stroke in PHC had been inadequate for this group of patients. According to the CHADS score, all the patients with atrial fibrillation who had visited PHC before the stroke occurred should have had some kind of intervention; but for a great proportion of these patients, no measures had been taken. Regarding the lifestyle-related risk factors smoking, alcoholism, and overweight/obesity, the proportion of patients treated should be seen in relation to the the very low rate of information about these risk factors in the medical records. This is alarming, especially since many previous studies have shown the benefits of treatment for modifiable risk factors in preventing stroke [4, 5]. 

Although the benefits of treating risk factors for cardiovascular disease (CVD) are well known, there is considerable interphysician variability [13, 14] in the application of this knowledge. The inadequate treatment of CVD risk factors in PHC could to some degree be due to such interphysician variability. Possible factors that could lead to general practitioners inadequate treatment of CVD risk factors in PHC may be due in part to a lack of time for each patient and reorganisations of the PHC system, resulting in poorer continuity for patients. A study from Norway found firm links between longitudinal, continuous care, and the general practitioners' accumulated knowledge about and sense of responsibility for their patients [15]. In addition, the authors of a study of the patients' own views on continuity in PHC found that having access to a personal doctor was positively associated with a sense of security [16], which in turn is probably related to better compliance with medication. Lack of compliance could sometimes discourage doctors in PHC from initiating measures in order to prevent stroke. For example, previous studies have shown that as many as 50% of all patients who receive medication for hypertension do not actually take the prescribed drug [17]. Principles of facilitating clinical negotiation with patients with chronic diabetes have been described in general practice in the UK. Some of the principles include patient centeredness, which can be achieved at a relatively low cost [18]. 

Before implementing large-scale, preventive interventions in PHC, it is of utmost importance to evaluate the cost-effectiveness of such interventions. A systematic review found that primary-care-based lifestyle interventions only resulted in small changes in behaviour, and none appeared to produce substantial changes [19]. A study from Southern England found that preventive care in patients with proven cardiovascular disease remains insufficient in general practice and concluded that: “As general practitioners we should ensure that we are providing high quality preventive care to patients with clinical disease before we focus on the even more demanding task of primary prevention” [20]. Five European countries and 754 randomly selected general practitioners were included in a study to investigate perceived obstacles in the implementation of guidelines for cardiovascular disease prevention. Key obstacles to a greater implementation of guidelines were lack of time, prescription costs, and patient compliance. Some suggestions for improving implementation were more education for both doctors and patients and simplifying the guidelines [21]. 

Effective primary prevention provided by general practitioners in PHC is associated with several obstacles and costs. It is possible that there should be more focus on other health care professionals in preventive work, such as district nurses. Many nurses have a high level of competence that could be used to improve detection of several risk factors for stroke. For example, a randomised trial found that secondary prevention led by nurses in PHC reduced future cardiovascular events and mortality by up to one third [22]. New strategies could also include more knowledge on the part of physicians concerning decision-making processes [23], as well as targeted screening for risk factors in the growing population of elderly people. However, more research is needed in order to create cost-effective interventions. 

### 4.1. Strengths and limitations

A major strength of this study was the inclusion of all important stroke risk factors and the thorough search for patients that included all medical records obtained from hospital registers and registers for outpatient care. Second, the validity of the CVD diagnosis using our approach was found to be high in an evaluation for the years 1987 and 1995 by the Swedish National Board of Health and Welfare [24]. Third, this study included all patients with first-ever stroke in a municipality in Stockholm that is representative for the rest of Sweden regarding socio-economic factors. Fourth, patients who were not hospitalised were also included although almost all patients with stroke are hospitalised in Sweden [25].

An important limitation is that data from medical records only provide information about what was recorded, and not about the actual intervention. In addition, the data in the medical records do not include enough information on whether initiated measures, such as drug therapy, also had an adequate effect. Therefore we could not evaluate how many of the patients treated for hypertension or diabetes actually reached the treatment goals. However, our findings indicate that initiated measures were carried out among an insufficient number of the patients. It is likely that even fewer patients reached the actual treatment goals. Second, data on cholesterol levels were not included in the study, since the use of medication for elevated cholesterol levels was not a part of the clinical practice in PHC at the time of the study. Third, since information on lifestyle factors is frequently not found in the records, our data probably underestimate the true occurrence of these risk factors. On the other hand the data probably overestimate interventions, because if measures are taken, it is more probable that information will be recorded.

## 5. Conclusions

This study showed that primary prevention of stroke in PHC can be improved, especially regarding atrial fibrillation and lifestyle-related risk factors. Health policies need to target not only the public, but also general practitioners and other health care professionals. New strategies could include more knowledge on the part of physicians regarding decision-making processes, as well as targeted screening for risk factors in the growing population of elderly people.

## Figures and Tables

**Table 1:** Known risk factors among 187 stroke patients and measures taken in primary health care for the 114 patients who had seen a general practitioner (GP) in the year preceding their stroke, in numbers and percentages.

Risk factor	Risk factors among all stroke patients^1^ (*n* = 187)	Risk factors among stroke patients *not* seen by a GP (*n* = 73)	Risk factors among stroke patients seen by a GP (*n* = 114)	Measures taken by a GP in patients with a risk factor^2^
Hypertension	78 (41.7%)	33 (45.2%)	45 (39.5%)	34 (75.6%)
Diabetes	42 (22.5%)	18 (24.7%)	24 (21.1%)	18 (75.0%)
Atrial fibrillation	43 (23.0%)	20 (27.4%)	23 (20.2%)	14 (60.9%)
Smoking^3^	44 (23.5%)	20 (38.5%)	24 (29.6%)	13 (54.1%)
Alcoholism^3^	17 (9.1%)	7 (28.0%)	10 (26.3%)	4 (40.0%)
Overweight/Obesity^3^	33 (17.6)	14 (63.6%)	19 (46.3%)	11 (57.9%)

^1^According to the medical records from all health care contacts.

^2^Denominator is the number of patients who had seen a general practitioner.

^3^The number of missing data for the lifestyle-related factors was high: 54 for smoking, 124 for alcoholism and 124 for overweight/obesity; the denominators have been adjusted accordingly.
